# A period prevalence study of palliative care need and provision in adult patients attending hospital-based dialysis units

**DOI:** 10.1007/s40620-024-02193-2

**Published:** 2025-01-27

**Authors:** Alannah L. Cooper, Natalie Panizza, Rebecca Bartlett, Dipna Martin-Robins, Janie A. Brown

**Affiliations:** 1 Royal Perth Hospital, Victoria Square, Perth, WA 6000 Australia; 2 School of Nursing, Curtin University, Kent Street, Bentley, WA 6102 Australia; 3 St John of God Midland Public and Private Hospital, 1 Clayton Street, Midland, WA 6056 Australia; 4 The Western Australian Group for Evidence Informed Healthcare Practice: A JBI Centre of Excellence, Curtin University, Kent Street, Bentley, Perth, WA 6102 Australia

**Keywords:** Palliative care, Life-limiting illness, Kidney disease, Haemodialysis

## Abstract

**Background:**

Advanced chronic kidney disease is a life-limiting disease that is known to benefit from palliative care. Unmet palliative care need in patients with kidney failure is commonly reported but the level of need among patients receiving haemodialysis is unknown.

**Methods:**

A period prevalence study of adult patients attending two hospital-based dialysis units was conducted. Patient medical records were reviewed using the Gold Standards Framework Proactive Indication Guidance to assess for potential palliative care need.

**Results:**

A total of 128 patient medical records were reviewed, 45% (*n* = 58) of patients could have potentially benefitted from palliative care. Of the patients with indicators for palliative care, 72% (*n* = 42) had no evidence of receiving or awaiting any form of palliative care. High levels of palliative care need were found in patients who identified as Aboriginal or Torres Strait Islander and non-Indigenous patients.

**Conclusions:**

This study found high levels of palliative care need among adult patients attending hospital-based dialysis units. The majority of patients with indicators were not receiving any form of palliative care.

**Graphical abstract:**

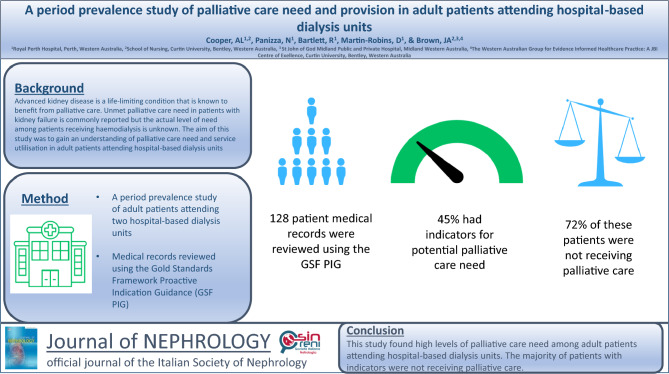

**Supplementary Information:**

The online version contains supplementary material available at 10.1007/s40620-024-02193-2.

## Introduction

Chronic kidney disease (CKD) is a progressive condition experienced by approximately 10% of the global adult population [1, 2]. Chronic kidney disease is defined as ‘… abnormalities of kidney structure or function, present for a minimum of three months, with implications for health’ [3]. The prevalence of CKD is increasing globally, and kidney disease is predicted to become the fifth leading cause of death by 2040 [4]. There are a wide range of factors that are associated with an increased risk of developing CKD including older age, ethnicity, lower socioeconomic status, hypertension, and diabetes mellitus [5].

In the Australian context, the most recently available data from the Australian Bureau of Statistics [6] determined that 11% of the adult population experienced CKD. The prevalence of CKD was 18% for Aboriginal and Torres Strait Islander adults. Aboriginal and Torres Strait Islander people were twice as likely as non-Indigenous people to experience CKD. As well as individual impacts, CKD places large demands on the Australian healthcare system. In 2020–21, 17% of all hospitalisations were related to a diagnosis of CKD and an estimated $1.9 billion of expenditure across the Australian healthcare system was due to CKD [7].

The timing and introduction of renal replacement therapy for kidney failure is individualised but is often initiated when symptoms such as fluid retention, hyperkalaemia, or uraemia become increasingly difficult to manage with medical therapies [8]. Kidney replacement therapy is achieved through transplantation or dialysis [9]. While transplantation is considered to be the optimal treatment option, not all patients are suitable candidates for transplantation, due to comorbidities, malignancy, age, or immunological status [8]. For patients who are not suitable for transplantation or those awaiting transplantation, dialysis can be used to support filtration of the blood and remove excess fluid, solutes, and toxins [10].

Generally speaking, peritoneal dialysis (PD) is favoured as the first line option for dialysis because patients are able to receive this treatment at home, have more autonomy and flexibility, require fewer hospital visits, and PD is also more cost-effective [11, 12]. Haemodialysis may be indicated for patients with contraindications for peritoneal dialysis and with more severe kidney failure [12] and is the most common type of dialysis received by people with kidney failure in Australia [7]. In 2021, 65% of people with kidney failure in Australia were receiving haemodialysis at satellite dialysis facilities, with 25% treated in hospital and 9% at home [7]. The requirements for satellite and hospital haemodialysis can be onerous for patients, requiring them to undergo haemodialysis typically three times a week, with each session lasting around four hours not including travel [8].

Understandably the requirements of in-hospital haemodialysis can significantly impact patient quality of life, and quality of life is lowest in CKD patients receiving haemodialysis [13]. While patients can receive dialysis for many years, the progressive nature of CKD means it is a life-limiting illness [8]. Kidney failure is recognised as a life-limiting illness that is advanced, incurable and is likely to lead to death [14]. Globally, CKD is a leading cause of death and is associated with reduced life expectancy [15]. Chronic kidney disease contributed to 12% of all deaths in Australia in 2021 [7]. Aboriginal and Torres Strait Islander people were four times more likely to die from CKD than non-Indigenous people. Deaths from CKD were also higher in men and people aged 85 or over. Internationally, countries with lower incomes have higher rates of mortality related to CKD compared to nations with higher incomes [1].

The quality of life of patients receiving maintenance haemodialysis is impacted by the high burden of psychological symptoms related to their ongoing treatments. This includes a high prevalence of anxiety and depression that negatively impacts patient’s mental health and quality of life [16]. Other psychological impacts include fluctuations in cognitive well-being across haemodialysis cycles, restrictions arising from the haemodialysis treatment schedule, and the emotional impact of haemodialysis on the self and others [17]. Psychological distress related to adjustment, death and dying, family and social functioning, and loss are also common amongst haemodialysis patients [18].

Individuals with kidney failure can also experience a multitude of physical symptoms that may include fatigue, mobility issues, pain, weight loss, heartburn, and poor sleep [13]. The symptom burden of fatigue, pain, and depression were found to be similar in patients with kidney failure and patients with gastrointestinal cancer [19]. These findings highlight the severity of disease burden that patients with kidney failure can experience. Despite acknowledgement of the importance of integrating advance care planning and palliative care discussions across the continuum of kidney failure, the evidence shows this is poorly implemented in practice [20, 21].

Haemodialysis patients have a similar symptom burden and prognosis to cancer patients but are more likely to die in hospital and are less likely to receive hospice care [22, 23]. In their final month of life, haemodialysis patients are also more likely to have admissions to an intensive care unit and receive invasive procedures, such as mechanical ventilation and resuscitation than patients with heart failure and cancer [24]. In recognition of the high symptom burden and poor prognosis that patients with kidney failure experience, many healthcare services have implemented kidney supportive care services. These services provide specialised support for symptom management and care planning for patients, with conservative management and patients receiving kidney replacement therapy [25]. While the models of kidney supportive care services reported in the literature vary, they are generally considered to be an integration of kidney and palliative medicine [26].

Chronic kidney disease is one of the 12 life-limiting conditions included in the Gold Standards Framework Proactive Identification Guidance (GSF PIG) [27] that are known to benefit from palliative care. The first iteration of the GSF PIG was developed in England in 2000 for community settings and has been refined and applied to other healthcare settings since its conception [28]. The GSF PIG provides indicators for palliative care for patients with stage 4 or 5 CKD whose condition is deteriorating, and assists clinicians to identify potential palliative care need. The integration of palliative care for patients receiving haemodialysis is associated with a reduced burden of physical symptoms and lower levels of depression [29]. Access to inpatient palliative care for patients with kidney failure has also been shown to reduce hospital readmissions for patients who were discharged, and to lower hospitalisation costs for patients who died in hospital [30].

Despite the significant morbidity associated with advanced CKD, integration of palliative care often occurs late or not all [31]. A study conducted in China found high levels of need and used a generalised tool to measure palliative care need that was not disease-specific [32]. Although this study investigated palliative care need in patients receiving haemodialysis, it was limited by its use of an assessment that did not include specific indicators for CKD as the primary determinant of potential need. While unmet palliative care need is identified as a common issue in patients with advanced CKD [33], the data describing the extent of need among patients receiving haemodialysis is limited. A more comprehensive understanding of the level of palliative care need is required to assist health services to facilitate optimal patient care.

## Methods

The Strengthening the Reporting of Observational Studies in Epidemiology (STROBE) (Supplementary File 1) was used to report the methodology and findings of this study.

### Aim

The aim of this study was to gain an understanding of palliative care need and service utilisation in adult patients attending hospital-based dialysis units. The objectives of the study were to:

1. Determine the size and characteristics of the population of adult dialysis patients with potential palliative care need at the study sites,
2. Establish what percentage of patients with potential palliative care need have been referred to and/or were receiving palliative care at the study sites,
3. Compare referral rates and access to palliative care service across study sites and palliative care service models.

### Design

A prospective period prevalence study was undertaken across the hospital-based dialysis units in the local health service. The design of this study is adapted from a previously reported inpatient point prevalence study and an outpatient period prevalence study [34, 35].

### Study setting

Study site 1 is a public metropolitan hospital with 470 beds offering a range of medical, surgical, and mental health services that sees approximately 80 dialysis patients per week. The dialysis units have eight stepdown chairs and 14 acute dialysis beds. There is a consultative model of specialist palliative care with current staffing levels of approximately 1.6 full-time equivalent provided by three Palliative Medicine Specialists Consultants, 1 full-time equivalent provided by Registrar, 1.8 full-time equivalent provided by two Nurse Practitioners, 1 full-time equivalent Clinical Nurse, 1 full-time equivalent Social Worker, and a non-clinical 1 full-time equivalent Secretary. Patients can be referred by their treating team to palliative care services and the palliative care team will visit these patients while they attend haemodialysis. There is a fortnightly clinic led by a palliative care Nurse Practitioner with specialisation in CKD for patients under conservative treatment. A monthly multi-disciplinary team meeting is held to review patients receiving haemodialysis. During 2023 there were 1583 referrals to the palliative care service.

Study site 2 is a public metropolitan hospital with a total of 211 beds for medical, surgical and mental health inpatients that sees approximately 50 dialysis patients per week. The dialysis unit has 12 dialysis beds. There is a consultative model of specialist palliative care with current staffing levels of approximately 0.2 full-time equivalent provided by one Palliative Medicine Specialist Consultant and 1 full-time equivalent provided by two Nurse Practitioners. There are no additional clinics or regular multidisciplinary team meetings that are attended by palliative care clinicians. During 2023 there were 495 referrals to the palliative care service.

### Sample and inclusion criteria

Adult dialysis patients who attended the study sites’ dialysis units during the nominated week of the period prevalence study were included. Patients who were < 18 years old, patients in the emergency department or intensive care unit, patients admitted on a medical or surgical ward or mental health ward, and patients admitted for a same day procedure such as day surgery were excluded from the study cohort.

### Data collection

Data were collected on all patients who attended the dialysis units during five nominated weekdays in March 2024. If a patient attended dialysis multiple times during the nominated period, data were only collected from their first visit. Two Registered Nurses performed data collection. They received training prior to commencing data collection to ensure consistency. A data dictionary (Supplementary File 2) was created to support data collection and provided clear definitions for the proactive indicators for palliative care for the 12 life-limiting conditions specified in the GSF PIG [27]. The GSF PIG aids early proactive identification of palliative care need drawing on the surprise question ‘Would you be surprised if the patient were to die in the next year, months, weeks, days?’ as well as indicators for 12 conditions [27]. The life-limiting conditions included in the GSF PIG are; cancer, chronic obstructive pulmonary disease (COPD), dementia, frailty, general neurological disease, heart disease, kidney disease, liver disease, motor neurone disease, multiple sclerosis, Parkinson’s disease, and stroke.

In the current study, criteria were established to determine if a patient could be appropriate for palliative care, drawing on the proactive indicators outlined for all 12 life-limiting conditions in the GSF PIG (Supplementary File 2). For the purposes of this study, evidence of palliative care need being met was defined as a palliative care approach from the treating team, such as appropriate use of Goals of Care, evidence of patient and or family discussion around future wishes, advance care planning to clearly define treatment ceilings, or referral to specialist palliative care services. Registered Nurses collected data from patient medical records and entered data directly into Qualtrics. Data were collected on participant characteristics and the 12 conditions that may benefit from palliative care outlined in the GSF PIG (Appendix 2).

### Data analysis

Data were exported to SPSS for analysis. Patient characteristics, level of palliative care need, and provision of palliative care are reported using descriptive statistics including frequencies and percentages. A Chi-Square test was used to compare and explore any difference in potential palliative care need based on identity.

## Results

Data were collected from128 medical records that were available for review across the two study sites. Of these, 76 attended dialysis at study site 1 and 52 attended dialysis at study site 2.

## Collective overview of the dialysis cohort

**Fig. 1:**
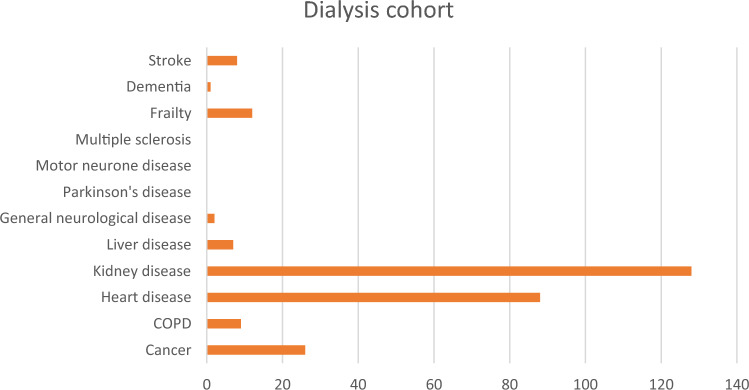
Prevalence of conditions listed in the Gold Standards Framework

The majority of patients were male (61%, n = 78) with an age range of 25 – 94 and a mean age of 63 years. The sample was predominately non-Indigenous (66%, n = 85); 33% (n = 63) of patients identified as Aboriginal and one patient (< 1%) as Torres Strait Islander. As well as experiencing kidney disease, the majority of patients (79%, n = 101) also experienced other life-limiting conditions outlined in the GSF PIG (Fig. 1). Cardiovascular morbidity was the most prevalent comorbidity experienced by patients.

### Assessment for potential palliative care need

**Fig. 2:**
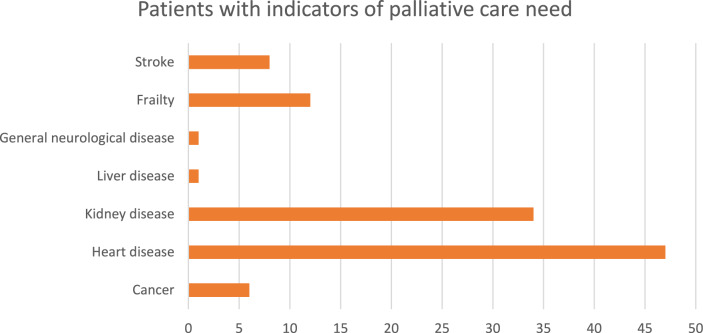
Prevalence of conditions with sufficient indicators of potential palliative care need

**Fig. 3:**
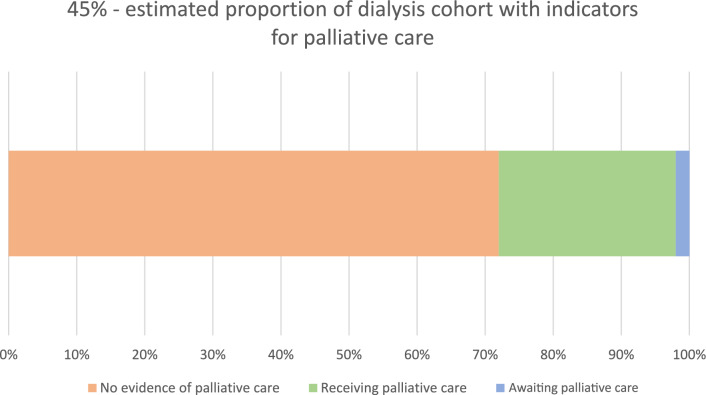
Prevalence of palliative care need and provision of palliative care in patients with indicators of potential palliative care need

All patients were assessed for potential palliative need based on the relevant indicators for the conditions each patient experienced in the GSF PIG. The response to the surprise question (would you be surprised if the patient were to die in the next year, months, weeks, days?) was “no” for 84% (n = 108) of patients. Based on the medical record review, 45% (n = 58) of dialysis patients could potentially benefit from palliative care. The number of conditions with the required number of positive indicators ranged from one to six for patients with potential palliative care need. The majority of patients (66%, n = 38) had more than one condition that had GSF PIG indicators for palliative care. Heart disease and kidney disease were the conditions that most often indicated palliative care need (Fig. 2). Of the 58 patients assessed as having indicators for palliative care, there was evidence of 26% (n = 15) currently receiving any form of palliative care, while 2% (n = 1) had been referred for specialist palliative care. For the majority of patients (72%, n = 42) there was no evidence of them receiving or awaiting any form of palliative care (Fig. 3). Of the 15 patients currently receiving palliative care, 87% (n = 13) were with involvement of specialist palliative care services and 13% (n = 2) of patients had evidence of a palliative care approach from their treating team.

### Comparison of potential palliative care need based on identity

Limited data regarding race, routinely collected as part of a patient’s health journey, were extracted and comparisons were made to see if there were any differences in the level of palliative care need between groups. The sample was predominately non-Indigenous 66% (n = 85); 33% (n = 63) of patients identified as Aboriginal and one patient (< 1%) as Torres Strait Islander. Patients who identified as Aboriginal or Torres Strait Islander were predominately female (56%, n = 24), with an age range of 25 to 89 and a mean age of 55. The majority of patients (84%, n = 36) who identified as Aboriginal or Torres Strait Islander experienced other life-limiting conditions listed in the GSF PIG as well as CKD. Non-Indigenous patients were predominately male (69%, n = 59), with an age range of 25 to 94 to and a mean age of 67. Most non-Indigenous patients (76%, n = 65) experienced at least one other condition listed in the GSF PIG (in addition to CKD). While both groups had patients with indicators of palliative care need and evidence of potential unmet palliative care, these were higher in patients who identified as Aboriginal or Torres Strait Islander (Table 1). Further analysis using a Chi-Square test found the level of unmet need between patients who identified as Aboriginal or Torres Strait Islander and those who identified as non-Indigenous was not statistically significant (*p* = 0.365).

**Table 1:** Comparison of level of need by race

	Total number of patients	Patients with indicators of palliative care need	Patients with indicators receiving palliative care
Aboriginal or Torres Strait Islander	*n* = 43	51% (*n* = 22)	18% (*n* = 4)
Non-Indigenous	*n* = 85	42% (*n* = 36)	31% (*n* = 11)^a^

Note. ^a^One patient had been referred to palliative care and was waiting to be seen

## Comparison of study sites

Given the differences in the size of the two study sites and the availability of palliative care resources, comparisons were made to assess the patient characteristics, level of palliative care need and provision of palliative care across the sites (Table 2).

**Table 2:** Comparison of patient characteristics and level of need across study sites

	Study site 1 (*n* = 76)	Study site 2 (*n* = 52)
Male	64% (*n* = 49)	56% (*n* = 29)
Mean age (range)	62 (30–90)	64 (25–94)
Identity		
Aboriginal	33% (*n* = 25)	33% (*n* = 17)
Non-Indigenous	66% (*n* = 50)	67% (*n* = 35)
Torres Strait Islander	1% (*n* = 1)	-
Patients with indicators of palliative need	42% (*n* = 32)	50% (*n* = 26)
Patients with indicators receiving palliative care	34% (*n* = 11)	15% (*n* = 4)

## Discussion

The aim of this study was to gain an understanding of palliative care need and service utilisation in adult patients attending hospital-based dialysis units. Across the two study sites an estimated 45% (n = 58) of the haemodialysis patient cohort could have potentially benefitted from palliative care, indicating a high level of need. This level of need is higher than in our previous cohorts of medical and surgical inpatients, where 29% of patients in a private hospital [35] had potential palliative care need. Although a private hospital population may differ, these findings highlight the similarity of disease burden for patients with kidney failure and cancer [19]; our earlier period prevalence that included oncology outpatients found 41% of patients had palliative care need [34].

Although the participant characteristics across the two sites were similar in terms of mean age, race, and proportion of patients experiencing other life-limiting illnesses, there was a higher proportion of patients at study site 2 with potential palliative care need compared to study site 1. As well as a higher proportion of potential need for palliative care in the study site 2 dialysis patient cohort, there was also a higher proportion of patients with indicators for palliative care with unmet need compared to study site 1. This could be reflective of the more limited palliative care services available at study site 2, and lack of integration of kidney and palliative medicine compared to study site 1 as a large tertiary hospital. A lack of supportive care services and palliative care integration are likely to result in worse physical and psychological symptoms and reduced quality of life for patients [13, 16]. Kidney supportive care services that integrate kidney and palliative care medicine have been shown to improve patient outcomes [29, 30] and are needed to optimise the care of patients with kidney failure.

The higher number of male patients receiving haemodialysis in the current study sample is in keeping with reports of the general population, where a higher incidence of kidney failure and need for haemodialysis is found in males [36, 37]. This provides confidence that the sample in this study is likely to be representative of the wider population of patients receiving haemodialysis. Although more women experience CKD, fewer progress to kidney failure. This may in part be due to the protective factors of oestrogen, but the reasons for this difference are not fully understood [38, 39]. The incidence of kidney failure by biological sex differs in Aboriginal and Torres Strait Islander people, with more females progressing to kidney failure and requiring dialysis compared to males [40]. This pattern, with a slightly higher predominance in female Aboriginal and Torres Strait Islander people, was also present in our sample, where 56% (n = 24) of Aboriginal and Torres Strait Islander people receiving haemodialysis were female.

Across the two study sites, a comparison of palliative care need for patients who identified as Aboriginal or Torres Strait Islander with non-Indigenous patients revealed that a higher proportion of Aboriginal or Torres Strait Islander patients had indicators for palliative care. There was also a higher proportion of Aboriginal and Torres Strait Islander people with unmet need compared to non-Indigenous patients. While not statistically significant, this difference reflects the increased disease burden of CKD experienced by Aboriginal and Torres Strait Islander people [36, 40] and the persistent issues with inequities and additional challenges that lead to gaps in care and poor health outcomes [40]. Co-designed interventions that are culturally sensitive need to be developed with Aboriginal and Torres Strait Islander people to identify and address their needs and improve access to supportive and palliative care.

The study only examines one metropolitan health service in Western Australia, so the generalisability of the results is limited and should be interpreted with this in mind. The results are also limited to patients receiving haemodialysis and do not represent patients receiving other forms of kidney replacement therapy. Data were collected based on the information available from patient medical records and assessed against the proactive indicators outlined in the GSF PIG. As the quality of information in medical records is variable, this may have resulted in under or over estimation of palliative care need.

## Conclusions

This study found high levels of palliative care need among adult patients attending hospital-based dialysis units. The majority of patients with indicators for palliative care were not receiving any form of palliative care. This represents a missed opportunity to provide optimal and holistic care for patients with kidney failure that is likely to result in a higher burden of symptoms and poorer quality of life. Interventions are needed to improve the provision of palliative care for patients with kidney failure who identify as Aboriginal or Torres Strait Islander people and for the broader population. Period and point prevalence studies can assist health services to determine the operational requirements needed to provide generalised and specialised palliative care for specific patient cohorts.

## Supplementary Information

Below is the link to the electronic supplementary material.

Supplementary file1 (DOCX 23 KB)

Supplementary file2 (DOC 83 KB)

## Data Availability

Data available on request from the authors.
