# Supplementary material for: A period prevalence study of palliative care need and provision in adult patients attending hospital-based dialysis units
Source: J Nephrol. 2025 Jan 27;38(2):687–95. doi: 10.1007/s40620-024-02193-2 (PMC11961506; doi:10.1007/s40620-024-02193-2)
Supplement: Supplementary file 1 — Supplementary file1 (DOCX 23 KB) [file 40620_2024_2193_MOESM1_ESM.docx]

**Supplementary file 2: Data dictionary**

## This data dictionary supports data collection for the research project entitled ‘A period prevalence study of palliative care need and provision in adult patients attending metropolitan hospital-based dialysis units’. Data were collected on all adult outpatients >18 years of age who attend the study site dialysis units on the five nominated days of the period prevalence study. Data were collected from the information available in patient’s medical records. The data collection tool is adapted from the Gold Standards Framework Proactive Identification Guidance (Thomas & Armstrong Wilson, 2016).

| **Item** | **Definition/ further information** |
| --- | --- |
| Patient UR number | Unique patient ID to be entered to ensure no duplication in data collection |
| Age | Record patients age in years (only patients who are >18 years of age are eligible) |
| Gender | Female or male or non-binary |
| Speciality the patient is being treated by | Please select the speciality the patient is being treated by e.g. nephrology |
| Does the patient have a history of any of the following conditions?   - Cancer - Chronic Obstructive Pulmonary Disease (COPD) - Heart disease - Renal disease - Liver disease - General Neurological disease - Parkinson’s disease - Motor Neurone disease - Multiple Sclerosis - Frailty - Stroke - Dementia | Check the patient’s medical notes for a history of the 12 conditions listed review based on the notes available up to a six-month timeframe for visits or admissions.  For patients with no history of these conditions the survey will end at this point.  For patients with any of these medical conditions further questions will be presented relevant to the conditions the patient has experienced. |
| Would you be surprised if the patient were to die in the next year, months, weeks, days? (use GSF flow chart to determine answer) | Use the Gold Standards Framework flowchart (below) to help guide your assessment of this question. Information on General Indicators of Decline and Specific Clinical Indicators can be found in the Gold Standards Framework Proactive Identification Guidance at the end of the data dictionary. |

**Condition specific questions**

| **Item** | **Definition/ further information** |
| --- | --- |
| If yes to cancer, does the patient have deteriorating performance status and functional ability due to metastatic cancer, multi-morbidities or not amenable to treatment? (**1 or more** items indicate palliative care need) | Based on review of patient medical notes select the criteria which are present or if none of these are present select ‘NONE of the above present’  Use ECOG to determine performance status from patient medical notes (see appendix at end of data dictionary). |
| If yes to cancer, presence of persistent symptoms despite optimal palliative management? | Yes or no based on review of patient medical notes |
| If yes to COPD, please tick all indicators which are present (**2 or more** indicate palliative care need) [ ] Recurrent hospital admissions (at least 3 in the last 12 months due to COPD)  [ ] MRC grade 4/5 – shortness of breath after 100 metres on the level or confined to house  [ ] Disease assessed to very severe (e.g. FEV1 <30% predicted), persistent symptoms despite optimal therapy, too unwell for surgery or pulmonary rehab  [ ] Fulfills long term oxygen therapy criteria (PaO2<7.3kPa)*  [ ] Required ICU/NIV during hospital admission  [ ] Other factors e.g. right heart failure, anorexia, cachexia, >6 steroids in preceding 6 months, requires palliative medication for breathlessness, still smoking | Review patient notes based on the available information select any indicators which are present – the presence of two or more indicate palliative care may be appropriate.  *Equivalent to PaO2 consistently <55mmHg May also be charted as oxygen saturation <88% in progress notes. Home oxygen in place or presence of oxygen referral form |
| If yes to Heart disease, please tick all indicators which are present (**2 or more** indicate palliative care need)  [ ] Patient for whom the surprise question is applicable  [ ] CHF NYHA Stage 3 or 4 with ongoing symptoms despite optimal HF therapy – shortness of breath on minimal exertion  [ ] Repeated admissions with heart failure – 3 admissions in 6 months or a single admission aged over 75 (50% 1yr mortality)  [ ] Difficult ongoing physical or psychological symptoms despite optimal tolerated therapy  [ ] Additional features include hyponatraemia <135mmol/l, high BP, declining renal function, anaemia, etc* | Review patient notes based on the available information select any indicators which are present – the presence of two or more indicate palliative care may be appropriate.  ***** Refractory to treatment/on last line treatment e.g. bumetimide |
| If yes to Kidney disease;  Does the patient have Stage 4 or 5 Chronic kidney disease? | Yes or no based on review of patient medical notes  If yes further questions will be generated to answer |
| If yes to stage 4 or 5 CKD, is the patient’s condition deteriorating please tick all indicators which are present (**2 or more** indicate palliative care need)  [ ] Patient for whom the surprise question is applicable  [ ] Repeated unplanned admissions (more than 3/year)  [ ] Patients with poor tolerance of dialysis with change of modality  [ ] Patients choosing the ‘no dialysis’ option (conservative), dialysis withdrawal or not opting for dialysis if transplant has failed  [ ] Difficult physical or psychological symptoms that have not responded to specific treatments  [ ] Symptomatic renal failure in patients who have chosen not to dialyse – nausea and vomiting, anorexia, pruritus, reduced functional status, intractable fluid overload | Review patient notes based on the available information select any indicators which are present – the presence of two or more indicate palliative care may be appropriate. |
| If yes to liver disease, are any of the following present? (**any one** indicates palliative care need)  [ ] Hepatocellular carcinoma  [ ] Liver transplant contra indicated  [ ] Advanced cirrhosis with complications including; refractory ascites, encephalopathy  [ ] Other adverse factors including malnutrition, severe comorbidities, hepatorenal syndrome  [ ] Bacterial infection current bleeds, raised INR, hyponatraemia, unless they are a candidate for liver transplant or amenable to treatment of an underlying condition | Review patient notes based on the available information select any indicators which are present – the presence of one or more indicate palliative care may be appropriate. |
| If yes to general neurological diseases, are any of the following present? (**any one** indicates palliative care need)  [ ] Progressive deterioration in physical and/or cognitive function despite optimal therapy  [ ] Symptoms which are complex and too difficult to control  [ ] Swallowing problems (dysphagia) leading to recurrent aspiration pneumonia, sepsis, breathlessness or respiratory failure  [ ] Speech problems: increasing difficulty in communications and progressive dysphasia | Review patient notes based on the available information select any indicators which are present – the presence of one or more indicate palliative care may be appropriate. |
| If yes to Parkinson’s disease, are any of the following present? (**any one** indicates palliative care need)  [ ] Drug treatment less effective or increasingly complex regime of drug treatments  [ ] Reduced independence, needs ADL help  [ ] The condition is less well controlled with increasing ‘off’ periods  [ ] Dyskinesias, mobility problems and falls  [ ] Psychiatric signs (depression, anxiety, hallucinations, psychosis)  [ ] Similar pattern to frailty* | Review patient notes based on the available information select any indicators which are present – the presence of one or more indicate palliative care may be appropriate.  *See Gold Standards Framework Proactive Identification Guidance |
| If yes to motor neurone disease, are any of the following present? (**any one** indicates palliative care need)  [ ] Marked rapid decline in physical status  [ ] First episode of aspirational pneumonia  [ ] Increased cognitive difficulties  [ ] Weight loss  [ ] Significant complex symptoms and medical complications  [ ] Low vital capacity (below 70% predicted spirometry), or initiation of NIV  [ ] Mobility problems and falls  [ ] Communication difficulties | Review patient notes based on the available information select any indicators which are present – the presence of one or more indicate palliative care may be appropriate. |
| If yes to multiple sclerosis, are any of the following present? (**any one** indicates palliative care need)  [ ] Significant complex symptoms and medical complications  [ ] Dysphagia and poor nutritional status  [ ] Communication difficulties e.g,. Dysarthria and fatigue  [ ] Cognitive impairment notably the onset of dementia | Review patient notes based on the available information select any indicators which are present – the presence of one or more indicate palliative care may be appropriate. |
| If yes to frailty, are any of the following present? (**any one** indicates palliative care need)  [ ] Patient for whom the surprise question is applicable  [ ] Multiple morbidities  [ ] Weakness, weight loss, exhaustion  [ ] At least three of the following; Aged over 85, Male, any health problems that limit activity, regularly uses a stick, walker or wheelchair to get about?  [ ] Frailty score 6-9 | Review patient notes based on the available information select any indicators which are present – the presence of one or more indicate palliative care may be appropriate. |
| If yes to dementia, are any of the following present? (**any one** indicates palliative care need)  [ ] Moderate or severe stage dementia  [ ] Unable to walk without assistance  [ ] Urinary and faecal incontinence  [ ] No consistently meaningful conversation  [ ] Unable to do ADLs  [ ] Clinical frailty score equal to or greater than 6  [ ] Plus any of the following; weight loss, urinary tract infection, severe pressure sores stage 3 or 4, recurrent fever, reduced oral intake, aspiration pneumonia. | Review patient notes based on the available information select any indicators which are present – the presence of one or more indicate palliative care may be appropriate. |
| If yes to dementia, does the patient have an advanced care plan in place? | Review patient notes is there an advance care plan in place? Yes/No |
| If yes to Stroke, are any of the following present? (**any one** indicates palliative care need)  [ ] Persistent vegetative, minimal conscious state or dense paralysis  [ ] Medical complications, or lack of improvement within 3 months of onset  [ ] Cognitive impairment / Post-stroke dementia  [ ] Other factors e.g. old age, male, heart disease, stroke sub-type, hyperglycaemia, dementia, renal failure | Review patient notes based on the available information select any indicators which are present – the presence of one or more indicate palliative care may be appropriate. |
| Based on your review could the patient potentially benefit from palliative care? | Did the patient meet any of the required thresholds which indicate palliative care may be appropriate? Yes/No  If no, the survey will end at this point. If yes survey will continue. |
| If the review indicated the patient could potentially benefit from palliative care, is the patient currently receiving palliative care? | Review patient notes to see if there is evidence of patient receiving palliative care. Yes/No |
| If yes to the previous question what type of palliative care is the patient receiving?  [ ] Specialist palliative care  [ ] Palliative care approach by treating team | Review patient notes to see what type of palliative care the patient is receiving.  Specialist palliative care is treatment by the palliative care team.  A palliative care approach by the treating team – is evidence of a palliative care approach by the patient’s usual treating team only (no input from palliative care services) and can include use of Goals of Care, comfort measures, stopping routine observations such as vital signs and changes to calling criteria related to palliation. |
| If no, is the patient currently receiving palliative care, has the patient been referred to palliative care services? | Review patient notes to see if there is evidence of palliative care referral being made. Yes/No |
